# Transitions from avoidance: Reinforcing competing behaviours reduces generalised avoidance in new contexts

**DOI:** 10.1177/1747021820943148

**Published:** 2020-08-21

**Authors:** Marc P Bennett, Bryan Roche, Simon Dymond, Frank Baeyens, Bram Vervliet, Dirk Hermans

**Affiliations:** 1 Medical Research Council, Cognition and Brain Sciences Unit, Cambridge, UK; 2 National University of Ireland, Maynooth, Ireland; 3 Swansea University, Swansea, UK; 4 KU Leuven, Leuven, Belgium

**Keywords:** Avoidance, generalisation, category-based generalisation, anxiety

## Abstract

Generalised avoidance behaviours are a common diagnostic feature of anxiety-related disorders and a barrier to affecting changes in anxiety during therapy. However, strategies to mitigate generalised avoidance are under-investigated. Even less attention is given to reducing the category-based generalisation of avoidance. We therefore investigated the potential of an operant-based approach. Specifically, it was examined whether reinforcing competing (non-avoidance) behaviours to threat-predictive cues would interfere with the expression of generalised avoidance. Using a matching-to-sample task, artificial stimulus categories were established using physically dissimilar nonsense shapes. A member of one category (conditioned stimulus; CS1) was then associated with an aversive outcome in an Acquisition context, unless an avoidance response was made. Next, competing behaviours were reinforced in response to the CS1 in new contexts. Finally, we tested for the generalisation of avoidance to another member of the stimulus category (generalisation stimulus; GS1) in both a Novel context and the Acquisition context. The selective generalisation of avoidance to GS1 was observed, but only in the Acquisition context. In the Novel context, the generalisation of avoidance to GSs was significantly reduced. A comparison group (Experiment 2), which did not learn any competing behaviours, avoided GS1 in both contexts. These findings suggest that reinforcing competing behavioural responses to threat-predictive cues can lead to reductions in generalised avoidance. This study is among the first study to demonstrate sustained reductions in generalised avoidance resulting from operant-based protocols, and the clinical and research implications are discussed.

Avoidance tendencies that persist in the absence of any physical or psychological threat can negatively impact psychosocial functioning and lead to psychopathology (Hayes et al., 1996; LeDoux et al., 2017; Vlaeyen & Linton, 2000, 2012). As such, problematic avoidance is a common diagnostic feature of anxiety-related disorders (Dymond & Roche, 2009; Krypotos et al., 2015; LeDoux et al., 2017). However, factors that both establish and weaken problematic avoidance are under-investigated.

Generalisation is a potential source of problematic avoidance. This describes a change in behaviour towards one or more stimuli/contexts due to an experience in which those stimuli/contexts were not featured (Boddez et al., 2017). For example, an individual with anxiety might avoid modes of transportation (e.g., a bus or train) after experiencing a traumatic ferry accident (Yule et al., 1990, 2000). Such avoidance stems from the spread of the effects of the direct conditioning history with an actual ferry to other related forms of travel and transportation via the conceptual or symbolic features of stimuli involved (Hayes & Hofmann, 2018). That is, conceptual information about threat-predictive cues are recruited during learning (“a ferry is form of transport”) such that the category itself is associated with threat (“transport is dangerous”) and its exemplars can spontaneously evoke avoidance (“buses are dangerous”) (Dunsmoor & Murphy, 2015).

For example, Dymond and colleagues (2011) established artificial stimulus categories using a matching-to-sample (MTS) task. An MTS task teaches participants, using corrective feedback, to group perceptually dissimilar stimuli like shapes and sounds (e.g., A goes with B and A goes with C) such that previously untrained combinations are formed (i.e., B goes with C, and vice versa). Afterwards, one category member was paired with an aversive sound/image (unconditioned stimulus; US) unless a key press avoidance response was made which postponed the aversive US. During a critical generalisation test stage, presentations of the other members of the threat-predictive category elicited heightened avoidance in the absence of any US, suggesting that category-based membership can indeed facilitate the spreading of avoidance behaviour (Dymond et al., 2018).

Generalised avoidance can be difficult to modify in clinical settings. This is an important challenge because avoidance tendencies undermine therapeutic opportunities to experience fear-relevant events as safe. To manage avoidance, exposure therapy typically relies on Pavlovian procedures that are also effective at reducing fear like extinction learning (Treanor & Barry, 2017). However, techniques derived from operant principles might be useful adjuncts to standard treatments because avoidance is a product of operant conditioning (Dinsmoor, 2001; Smith et al., 2020). One operant-based approach to reduce avoidance might be to reinforce competing and incompatible behaviours (Petscher et al., 2009; Poling & Ryan, 1982). Shifting the delivery of rewards to favour competing classes of behaviour is commonly found to lower the probability of another target behaviour (Vollmer & Iwata, 1992). That is, declines in problem behaviour are observed once new classes of competing behaviours are strengthened. These techniques often feature in Applied Behavioural Analysis (ABA) therapies to attenuate aggressive and/or disruptive behaviours that are routed in schedules of negative reinforcement. As a simple example, Durand and Carr (1991) identified students (diagnosed with neuro-developmental delay) whose problem behaviours were maintained by the escape from academic demands. These authors demonstrated that selectively reinforcing alternative behavioural strategies to seek attention and academic support resulted in significant reductions in problem behaviours. This is an example of a Differential Reinforcement of Alternative (DRA) protocol (Durand & Carr, 1991).

Across two experiments, this study investigated whether the generalisation of avoidance across Novel contexts is reduced after the differential reinforcement of competing behaviours. There were four phases. First, perceptually dissimilar shapes were grouped through feedback into artificial categories using an MTS task (e.g., Category 1 [CAT1] = X1-CS1-GS2; Category 2 [CAT2] = X2-CS2-GS2). Second, a member of one category (conditioned stimulus; CS1) was paired with an aversive US unless an avoidance response was made (in the Acquisition context). Third, competing behaviours to CS1 were reinforced in new contexts. These new contexts functioned as “occasion setters” that signalled the availability of a reinforcer given the production of a particular competing behaviour in response to CS1. Specifically, pressing new buttons resulted in evaluative feedback (in Context 1 and/or Context 2). Finally, generalisation of avoidance to the other members of the stimulus categories (generalisation stimulus; GS1) was tested in the original Acquisition and a new Novel context. Avoidance was measured relative to a within-subject control category that was not associated with the US or avoidance.

We predicted that because a threat-predictive stimulus (CS1) was associated with competing behaviours in new contexts, the GS1 would evoke less avoidance in the Novel context relative to the Acquisition context. We also assumed that decreases in avoidance would result from learning competing behaviours. To test this, two groups were recruited. One group completed extended training—these participants learned two sets of competing behaviours across two contexts (in Context 1 and Context 2). A second group completed limited training—these participants learned just one set of competing behaviours in one context (in Context 1 only). As the Limited Training group learned fewer competitive behaviours, we expected this group to produce relatively more generalised avoidance in the Novel context.

## Experiment 1

### Method

#### Participants

Volunteers were recruited from a university recruitment pool. Individuals were excluded if they self-reported blood phobia and/or auditory sensitivity and if they already participated in similar research. Thirty-five participants were recruited (*M* = 21.54 years, *SD* = 4.88; 30 females) and compensated with course credit or €8. The Social and Societal Ethical Committee of KU Leuven approved this study, and all participants provided written informed consent.

#### Setting and stimuli

**Figure 1.**
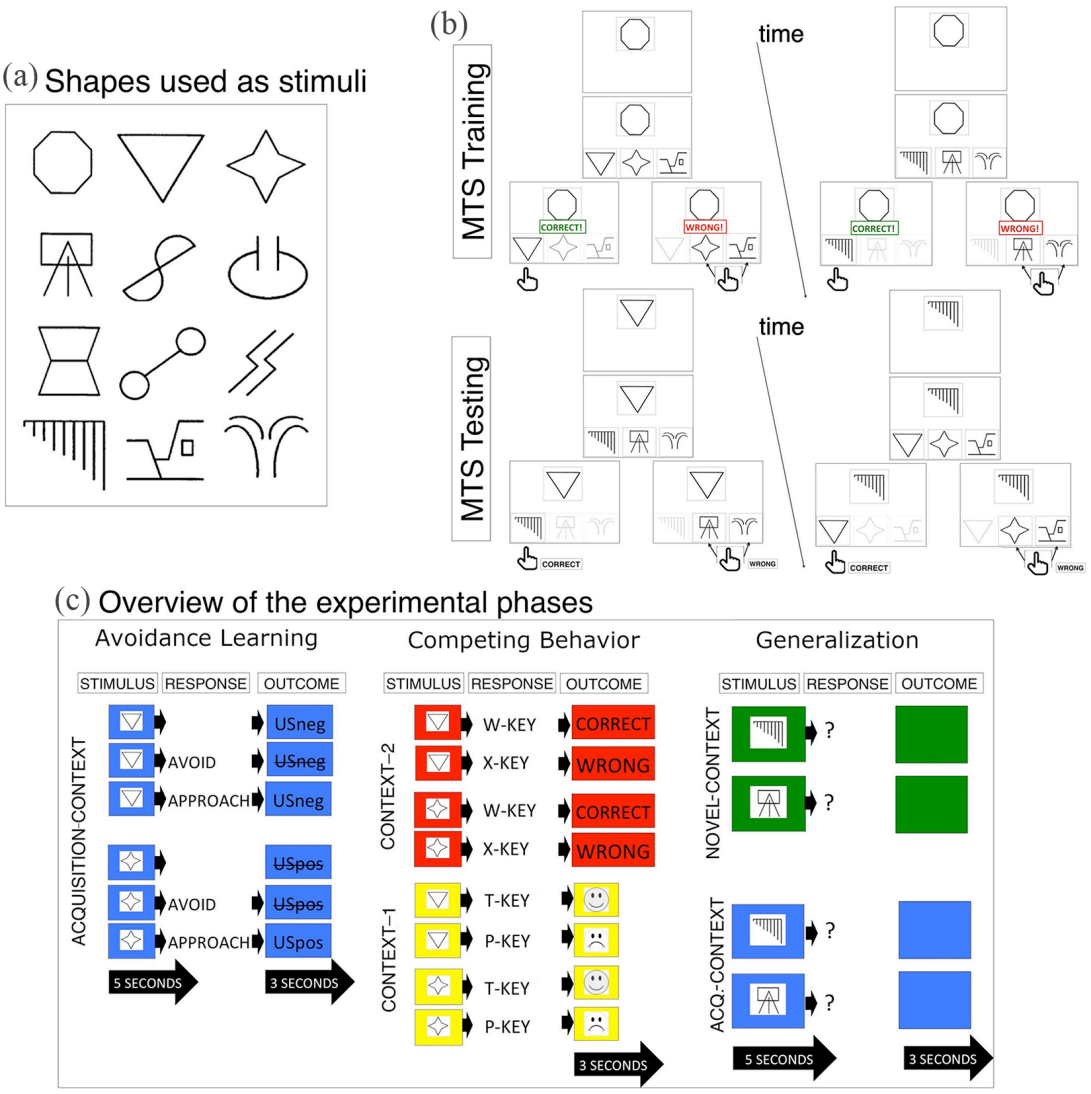
Schematic overview of the experimental stimuli and stages. (a) Stimuli: Physically dissimilar shapes were randomly assigned as conditioned stimuli (CSs) and generalisation stimuli (GSs). (b) Category learning: Two stimulus categories were established using a matching-to-sample (MTS) task. In a block of MTS training trials, relating CS1 and GS1 with X1 was reinforced using corrective feedback. Relating CS2 and GS2 with X2 was also reinforced. A block of testing trials then probed participants related CS1 with GS1 and CS2 with GS2, in the absence of any corrective feedback. (c) Experimental stages: In the Acquisition context, avoidance of CS1 and approach of CS2 were reinforced. Competing behaviours were then reinforced in response to the CSs. One group completed extended training—these participants learned two sets of competing behaviours across two contexts. A second group completed limited training—these participants learned just one set of competing behaviours in one context. We then tested for generalised avoidance. Here, GS1 and GS2 were presented in a Novel context and the Acquisition context.

Experimental sessions were conducted inside sound-attenuated cubicles. Stimuli were presented using a Microsoft Windows XP (Dell Optiplex 755) and a 17″ monitor (1024 × 768 pixels). Stimulus presentation and response recordings were programmed using Affect 4.0 (Spruyt et al., 2010). Abstract shapes were grouped into two stimulus categories (160 × 160 pixels; Figure 1). These stimuli are referred to below as X1, X2, CS1, CS2, CS3, GS1, GS2, and GS3. Context was cued using background colours (red, blue, yellow, and green) (Figure 1). These context cues are referred to below as Acquisition context, Context 1, Context 2, and Novel context. Stimuli and context were arranged into four different counterbalances. Participants were randomly assigned to these counterbalances prior to testing. These counterbalances had no effect on any dependent variable, so are not described further.

Avoidance was established using a negative valence US (USneg) validated previously in our laboratory (Bennett, Hermans, et al., 2015; Bennett, Vervoort, et al., 2015; Dymond et al., 2011; Lenaert et al., 2014). This involved a combination of an unpleasant image and sound. One of 12 body mutilation images from the International Affective Picture System (IAPS) was randomly shown for 3 s (1024 × 768 pixels) (Lang et al., 2008; Supplementary Materials). Simultaneously, a female scream was played via headphones for 2 s at 90 dB. Approach was also motivated using a positive valence US. This involved a 3-s message reading “Good! + 10 points! You now have [x + 10] points.” This US was based on unreported pilot research. We found that while negative images/sounds motivated avoidance learning, positive images/sounds were poorer motivators of approach tendencies relative to arbitrary points. Stimuli and software are available online (osf.io/rhfx7/). The exact task instructions are also outlined in Supplementary Materials.

##### Procedure

Figure 1 illustrates the four experimental stages: (1) category learning, (2) avoidance learning, (3) differential reinforcement of competing behaviours, and (4) generalised avoidance test. These were completed in order and without a break. Automated instructions appeared onscreen between each stage (Supplementary Materials). Sessions lasted approximately 45 min.

1. Category learning

Participants completed an MTS task and were instructed to group shapes by matching a shape from the lower screen with one at the upper screen (Figure 1b). On each MTS training trial, a sample stimulus appeared at the upper screen ([X1] or [X2]). Comparison stimuli appeared 500 ms later at the lower screen ([CS1, CS2, CS3] or [GS1, GS2, GS3]). Participants grouped a comparison stimulus with the sample via key presses (1 key = left comparison, 2 key = middle comparison, and 3 key = right comparison). Corrective feedback appeared for 2 s after each selection (the word “Correct” or “Wrong”). There were four types of training trials: [X1] → [**CS1**, CS2, CS3], [X2] → [CS1, **CS2**, CS3], [X1] → [**GS1**, GS2, GS3], and [X2] → [GS1, **GS2**, GS3] (correct option in bold). Training trials appeared pseudo-randomly (no more than two consecutive trial types) until 16 consecutively correct answers were made. Trials were separated by a 1- to 2-s inter-trial interval (ITI).

On each MTS testing trial, a stimulus was presented on the upper screen. Comparison stimuli appeared 500 ms later at the lower screen. Participants grouped stimuli using the same key presses. There were four test trial types: [CS1] → [**GS1**, GS2, GS3], [CS2] → [GS1, **GS2**, GS3], [GS1] → [**CS1**, CS2, CS3], and [GS2] → [CS1, **CS2**, CS3] (correct option in bold). Each trial appeared four times in a single testing block. No corrective feedback was provided. Test trials established whether participants could spontaneously group comparison stimuli based on a common sample (CS1 with GS1 / CS2 with GS2). Thus, two stimulus categories were established ([CAT1 = X1-CS1-GS1] and [CAT2 = X2-CS2-GS2]).

2. Avoidance learning

Instructions stated that USneg or USpos (positive valence US) might follow the shapes. Afterwards, the screen changed to the Acquisition context (e.g., blue background). There were 12 Pavlovian conditioning trials using a partial reinforcement schedule. On five Pavlovian trials, CS1 appeared for 5 s and was followed by USneg for 3 s. On one Pavlovian trial, CS1 appeared for 5 s but was not followed by USneg. On five Pavlovian trials, CS2 appeared for 5 s and was followed by USpos for 3 s. On one Pavlovian trial, CS2 appeared for 5 s but was not followed by USpos. Trials appeared pseudo-randomly and were separated by 1–2 s (ITI).

Instructions then appeared and directed participants to learn, through trial-and-error, to avoid the USneg (or access the USpos) by pressing either the spacebar or the return key. Afterwards, the screen colour changed to the Acquisition context and avoidance learning trials began. On each avoidance learning trial, a CS appeared for 5 s when key pressing was recorded. Spacebar presses omitted the pending USs, while return key presses triggered USs. The following contingencies describe CS1 trials (Figure 1): If no response was recorded in response to CS1, then CS1 was followed by a 3-s USneg. If a spacebar press was made to CS1, then the USneg was cancelled (this provided our measure of active USneg avoidance). If a return key press was made to CS21, then CS1 was followed by a 3-s USneg (USneg approach). The following contingencies describe CS2 trials (Figure 1b): If no response was recorded in response to CS2, then it was not followed by a 3-s USpos. If a return key press was made to CS2, then it was followed by a 3-s USpos (this provided our measure of active USpos approach). If a spacebar press was made to CS2, then it was not followed by the USpos. Avoidance trials continued pseudo-randomly until the USneg was avoided on six trials.

3. Differential reinforcement of competing behaviours

Competing behaviours were trained in new contexts. Instructions stated that the task was to learn new responses via trial-and-error. The between-groups factor was introduced at this stage—the extended or limited training of competing behaviours.

3A. *Extended training*. Competing behaviour training trials were presented across two blocks: a block of Context 1 trials and a block of Context 2 trials. The order of these blocks was randomized across participants. Prior to the block of Context 1 trials, instructions directed participants to use the T and P keys. The screen colour then changed to Context 1 (e.g., red screen). On each trial, a CS appeared and was replaced with 2 s written feedback (“correct” or “wrong”) once a key press was recorded. T key presses to CS1 and P key presses to CS2 were followed by “correct.” Errors were followed by “wrong.” Prior to the block of Context 2 trials, instructions directed participants to use the W and X keys. On each trial, a CS appeared and was replaced with 2 s visual feedback (smiling or frowning emoticon) once a key press was recorded. W key presses to CS1 or X key presses to CS2 were followed by a smiling emoticon. Errors were followed by a frowning emoticon. Trials appeared pseudo-randomly until six consecutively correct responses were made.
3B. *Limited training*. Participants in this group completed two blocks of Context 1 training trials.
3C. *Accuracy check*. A block of competing behaviour testing trials was administered. For the Extended Training group, CSs appeared randomly in the Acquisition context, Context 1, or Context 2. For the Limited Training group, CSs appeared randomly in the Acquisition context or Context 1. The same stimulus–response contingencies as reported above applied. This is summarized here: For CS1 trials, (1) avoidance was reinforced in the Acquisition context, (2) T key presses were reinforced in Context 1, and (3) W key presses were reinforced in Context 2. For CS2 trials, (1) approach was reinforced in the Acquisition context, (2) P key presses were reinforced in Context 1, and (3) X key presses were reinforced in Context 2. Trials continued until 36 consecutively correct responses were made. Corrective feedback was presented on only 50% of trials.

4. Test for generalised avoidance

Participants were instructed that their task was to now make whichever response they thought was most appropriate. Generalised avoidance was tested across two blocks. First, GS1 and GS2 were randomly presented four times each in the Novel context (e.g., green screen). Second, GSs were randomly presented four times each in the Acquisition context. On each of the trials, a GS appeared for 5 s, and all key presses were recorded. The GS was then terminated if a response was made within 5 s. Responses were not followed by any outcomes (Figure 1c).

#### Data analysis strategy

##### Manipulation checks

Three criteria were checked. First, it was tested whether artificial stimulus categories were established. The percentage of correct responses on MTS training trials and testing trials was calculated. Second, it was tested whether avoidance was heightened for CS1 relative to CS2. A repeated measures analysis of variance (RM-ANOVA) compared the percentage of avoidance responses to CS1 and CS2 during avoidance learning trials. In addition, RM-ANOVA examined whether approach responses were heightened for CS2 relative to CS1. Third, it was to test whether competing behaviours were learned. The percentage of correct trials during the competing behaviour training and testing trials was calculated. In each of the above ANOVA models, group was included as a between-group factor to test for any prior differences between the training conditions.

##### Outcome measures

It was predicted that GS1 would elicit heightened avoidance relative to GS2 in the Acquisition context, but not in the Novel context (i.e., a Stimulus × Context interaction). We also predicted this effect to be greater in the Extended Training group relative to the Limited Training group (i.e., a Stimulus × Context × Group interaction). Avoidance responses (i.e., spacebar press) to GS1 and GS2 were counted in both contexts. An RM-ANOVA was calculated with stimulus (GS1 vs. GS2) and context (Acquisition context vs. Novel context) as within-subjects factors and group (Extended vs. Limited training) as a between-group factor. Similar RM-ANOVAs were calculated to examine the effect of stimulus (GS1 vs. GS2), context (Acquisition context vs. Novel context), and group (Extended vs. Limited training) on (1) competing behaviours (i.e., T, P, X, and W keys) and (2) approach behaviours (i.e., return key). However, there were no specific predictions for these outcome measures as the focus of this study was on avoidance responding.

For all RM-ANOVAs, Greenhouse–Geisser correction is reported when Mauchly’s test could not assume sphericity. Effect sizes were calculated using partial eta squared ($\eta_{p}^{2}$). For all post hoc tests, alpha thresholds were corrected for multiple comparisons using Bonferroni corrections. Raw data and processing scripts are available online at osf.io/rhfx7/. All main effects and interaction effects are described in Supplementary Materials (Tables S1 to S3). The mean rates of avoidance, approach, and competing behaviour in response to each GS during the generalisation tests (and for each group) are also illustrated in Supplementary Materials (Figures S1 and S2).

### Results

#### Manipulation checks

##### Category learning

MTS task training and testing trials were completed with a high level of accuracy (training accuracy >86%; testing accuracy >88%) (Table 1). Also, the between-group effects were non-significant (Table 1). This suggests that two stimulus categories were established; CS1 was categorically related to GS1 and CS2 was categorically related to GS2.

**Table 1.** Response accuracy during category learning and competing behaviour learning stages in Experiment 1.

Accuracy	Total		Training group				Effect of group	
Category learning	*M*	*SD*	Extended		Limited		*F*	*p*
			*M*	*SD*	*M*	*SD*		
Training (%)	86.74	7.51	86.86	8.90	86.61	5.96	0.01	.92
Testing (%)	91.43	18.69	94.44	17.66	88.23	19.75	0.96	.33
Competing behaviour								
Training (%)	95.16	4.08	94.60	3.17	95.75	4.90	0.69	.41
Testing (%)	96.95	4.99	96.40	5.53	97.53	4.44	0.44	.51

##### Avoidance learning

**Figure 2.**
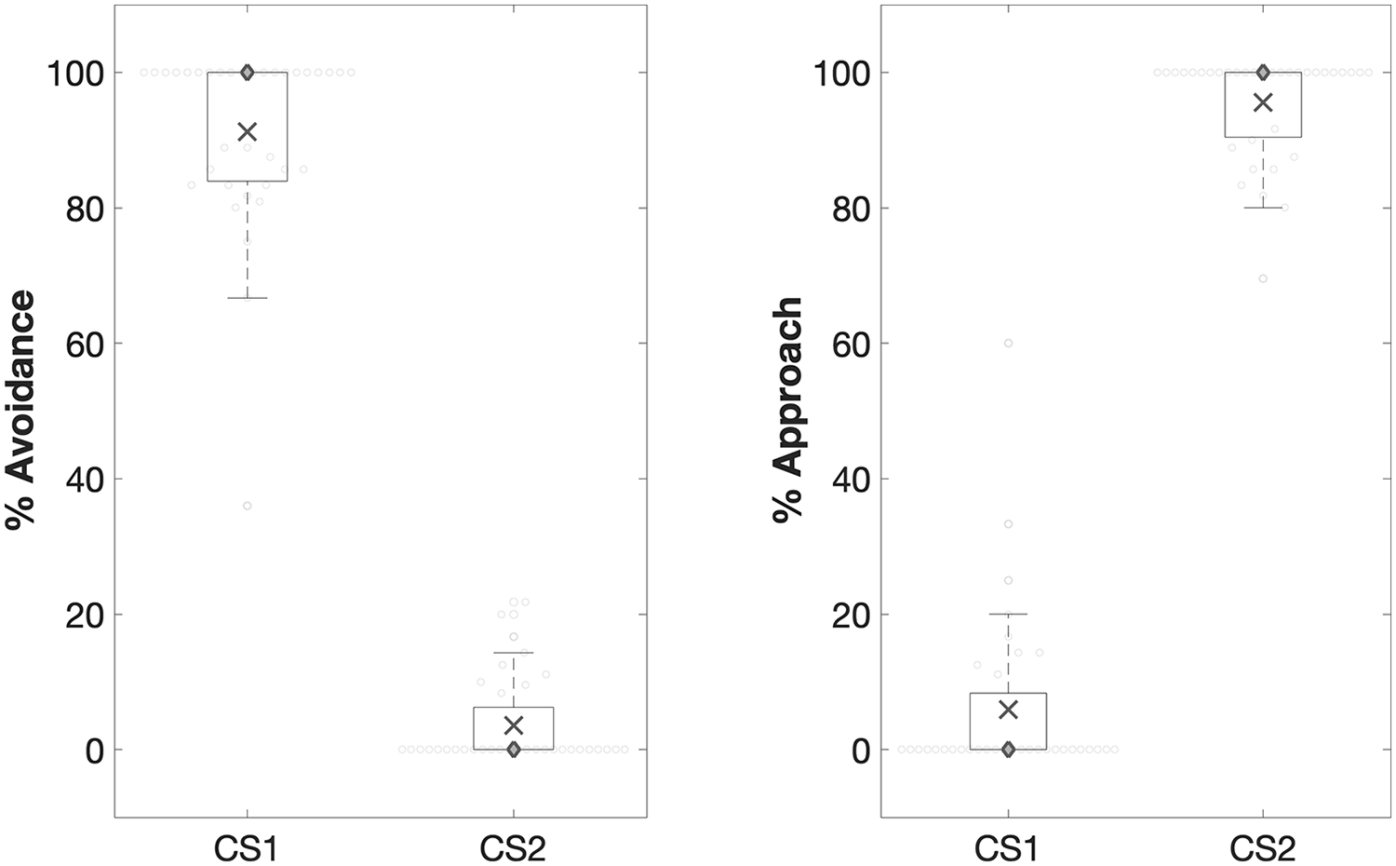
*Avoidance learning*. CS1 triggered more avoidance than CS2 during avoidance learning. CS2 triggered more approach than CS1. ○ = Individual data points; X = mean; ◊ = median. Edges of the box are the 25th and 75th percentiles. Whiskers extend to extreme value not considered to be an outlier: 2.7th and 99.3th percentile (based on MATLAB’s boxplot function). ***p < .0001.

Avoidance was heightened for CS1 relative to CS2, *F*(1, 33) = 815.01, *p* < .001, $\eta_{p}^{2}=.96$ (Figure 2). There was a non-significant effect of group on avoidance, *F* < 1, *p* = .53, and a non-significant two-way interaction between group and stimulus, *F* < 1, *p* = .83. Also, approach was heightened for CS2 relative to CS1, *F*(1, 33) = 762.37, *p* < .001, $\eta_{p}^{2}=.96$. Again, there was a non-significant group effect, *F* < 1, *p* = .70, and a non-significant two-way interaction between stimulus and group, *F* < 1, *p* = .77 (Figure 2).

##### Differential reinforcement of competing behaviours

Competing behaviour training and testing trials were completed with a high level of accuracy (training accuracy >94%; testing accuracy >96%), and the between-group effects were non-significant (Table 1). This suggests that competing behaviours in response to CS1 and CS2 were learned.

#### Outcome measures

##### Generalisation of avoidance

**Figure 3.**
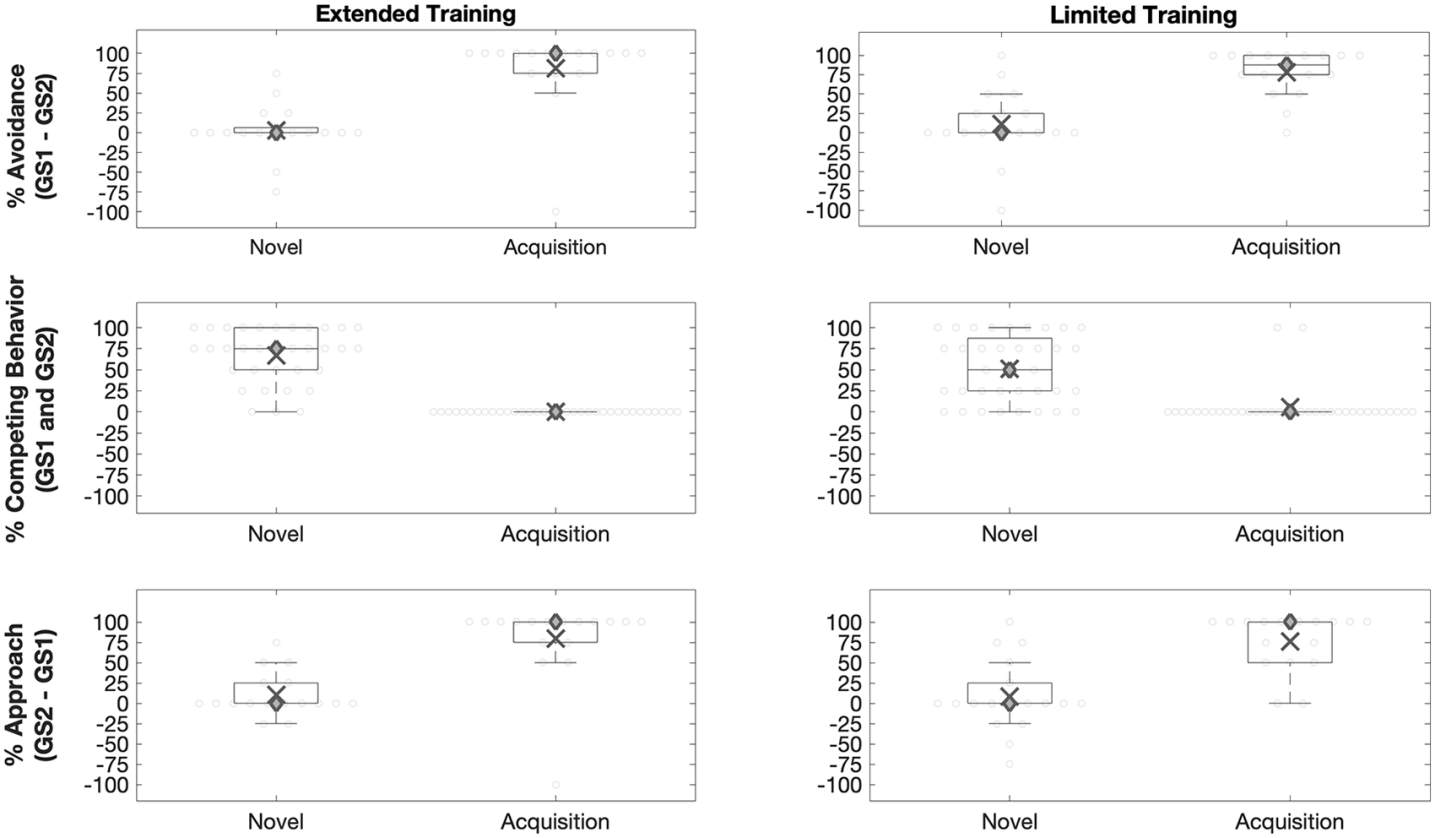
*Outcome measures: Generalised avoidance*. Generalised avoidance was estimated as responding to GS1 relative to GS2. A positive score indicates more avoidance of GS1. A negative score indicates more avoidance of GS2. Relative avoidance of GS1 was greater in the Acquisition context than in the Novel context. This was evident in both training groups. *Competing behaviours*. There was no effect of stimulus on competing behaviour. Overall, competing behaviours were more frequent in the Novel context than in the Acquisition context. *Generalised approach*. Generalised approach was estimated as responding to GS2 relative to GS1. A positive score indicates more approach of GS2. A negative score indicates more approach of GS1. Relative approach of GS2 was greater in the Acquisition context than in the Novel context. This was evident in both groups. ○ = Individual data points; X = mean; ◊ = median. Edges of the box are the 25th and 75th percentiles. Whiskers extend to extreme value not considered to be an outlier.

An RM-ANOVA examined the effect of stimulus, context, and group on avoidance. GS1 was predicted to elicit heightened avoidance relative to GS2 in the Acquisition context, but not in the Novel context. This was supported by a significant stimulus by context interaction, *F*(1, 33) = 100.80, *p* < .001, $\eta_{p}^{2}=.75$ (Table S1). Post hoc tests also revealed that avoidance of GS1 (relative to GS2) was greater in the Acquisition context than in the Novel context, *t*(17) = 6.83, *p* < .001 (Figure 3). The Extended Training group was expected to produce greater reductions in generalised avoidance. However, there was a non-significant effect of group, *F* < 1, *p* = .65, and a non-significant three-way interaction between group, stimulus, and context, *F* < 1, *p* = .44. This finding suggests that there was no difference in the impact of training group (Limited vs. Extended) on the observed reduction of avoidance.

##### Competing behaviours

**Figure 4.**
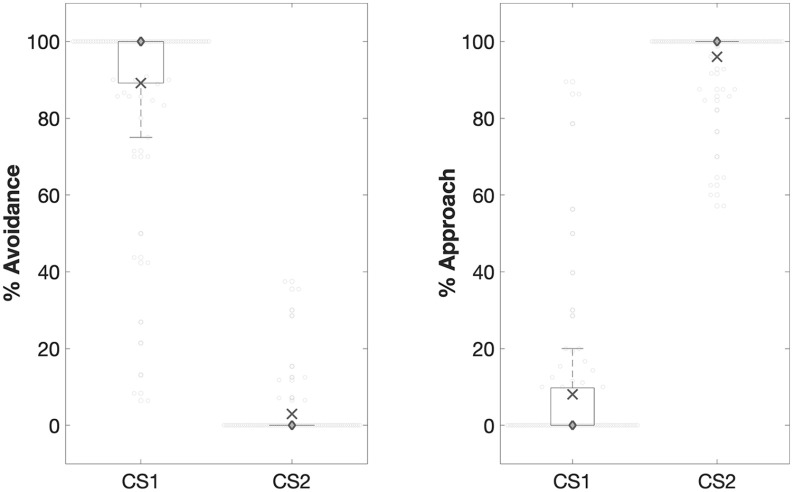
*Avoidance learning*. CS1 triggered more avoidance than CS2 during avoidance learning. CS2 triggered more approach than CS1. ○ = Individual data points; X = mean; ◊ = median. Edges of the box are the 25th and 75th percentiles. Whiskers extend to extreme value not considered to be an outlier. The absence of a box indicates that the 25th and 75th percentiles overlapped with the median value. **p < .0001.

An RM-ANOVA examined the effect of stimulus, context, and group on competing behaviours. We had no prior predictions, but competing behaviours could be expected to be more frequent in the Novel context relative to the Acquisition context. This was supported by a significant effect of context on competing behaviours, *F*(1, 33) = 69.48, *p* < .001, $\eta_{p}^{2}=.68$ (Figure 4; Table S2). In addition, competing behaviours in response to GS1 and GS2 did not differ. This was indicated by a non-significant effect of stimulus, *F* < 1, *p* = .66, and a non-significant interaction between stimulus and context, *F* < 1, *p* = .66. The Extended Training group might have been expected to produce a greater number of competing behaviours because they learned more of them. However, there was a non-significant main effect of group on competing behaviours, *F* < 1, *p* = .39, and all group interaction effects were non-significant (Table S2).

##### Approach behaviour

**Figure 5.**
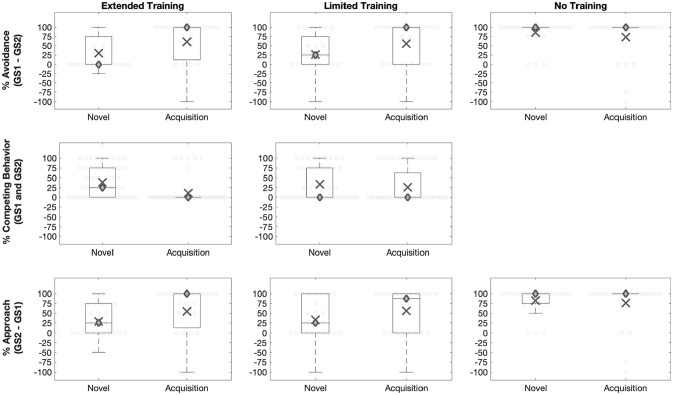
*Outcome Measures: Generalised avoidance*. Generalised avoidance was estimated as responding to GS1 relative to GS2. A positive score indicates more avoidance of GS1. A negative score indicates more avoidance of GS2. In the Extended and Limited Training groups, relative avoidance of GS1 was greater in the Acquisition context than in the Novel context. In the No-Training group, relative avoidance of GS1 did not differ between the Novel context and the Acquisition context. *Competing behaviours*. There was no effect of stimulus on competing behaviour. Overall, competing behaviours were more frequent in the Novel context than in the Acquisition context. *Generalised approach*. Generalised approach was estimated as responding to GS2 relative to GS1. A positive score indicates more approach of GS2. A negative score indicates more approach of GS1. In the Extended and Limited Training groups, relative approach of GS2 was greater in the Acquisition context than in the Novel context. In the No-Training group, relative avoidance of GS2 did not differ between the Novel context and the Acquisition context. ○ = Individual data points; X = mean; ◊ = median. Edges of the box are the 25th and 75th percentiles. Whiskers extend to extreme value not considered to be an outlier. The absence of a box indicates that the 25th and 75th percentiles overlapped with the median value.

An RM-ANOVA examined the effect of stimulus, context, and group on approach behaviour. There was a significant interaction between stimulus and context, *F*(1, 33) = 56.90, *p* < .001, $\eta_{p}^{2}=.63$ (Table S3). Approach of GS2, relative to GS1, was greater in the Acquisition context than in the Novel context, *t*(17) = 5.63, *p* < .001 (Figure 5). This indicates that generalised approach in response to GS2 was reduced in a Novel context. There was no main effect of group, *F* < 1, *p* = .76, and no three-way interaction between group, context, and stimulus, *F* < 1, *p* = .95. This suggests that there was no difference in the impact of training group on the observed reduction of approach.

### Discussion

In the Acquisition context, a threat-predictive stimulus (CS1) was associated with avoidance. Competing behaviours were reinforced in response to this threat-predictive stimulus in different contexts. Afterwards, categorically related stimuli (GS1) were found to elicit relatively less avoidance in Novel contexts than in the original Acquisition context. These findings suggest that the differential reinforcement of competing behaviours might be a useful technique to mitigate generalised avoidance.

It was assumed that any reductions in avoidance resulted from the reinforcement of competing behaviours. To test this, one group learned more competing behaviours than another; it was expected that the former group would produce greater reductions in avoidance. This was not the case. Therefore, an alternative explanation for reduced avoidance might simply be that presenting generalisation stimuli in a Novel context disrupted avoidance. To further clarify this, Experiment 2 included a third comparison group that did not learn any competing behaviours.

## Experiment 2

### Method

#### Participants, stimuli, and settings

Seventy-nine participants (66 females) were recruited (*M* = 20.17 years, *SD* = 4.94) and compensated with course credit or €8. All were fluent Flemish speakers and undergraduate students. The same exclusion criteria from Experiment 1 were applied. Stimuli, settings, and task instructions were identical to those reported in Experiment 1. Participants were randomly assigned to an Extended Training group (*n* = 27), a Limited Training group (*n* = 26) or a No-Training group (*n* = 26). Task, software, data, and scripts are available online (osf.io/rhfx7/).

#### Procedure

The four experimental stages were identical to those reported in Experiment 1. (1) Category learning: stimuli were grouped into two artificial categories using an MTS task ([CAT1 = X1-CS1-CS2] and [CAT2 = X2-CS2-CS2]). (2) Avoidance learning: a member of one category (CS1) was paired with an aversive US unless an avoidance response was made. A within-subject control stimulus (CS2) was not associated with avoidance. (3) Differential reinforcement of competing behaviours: Three groups were recruited in Experiment 2. The Extended Training group (*n* = 26) and the Limited Training group (*n* = 26) were identical to Experiment 1. A third comparison group was included that did not learn any competing behaviours (*n* = 26). This is referred to as the No-Training group. These participants automatically transitioned from Stage 2 of the experiment to Stage 4. (4) Test for generalised avoidance: responding to generalisation stimuli (GS1 and GS2) was tested in a Novel context and the Acquisition context.

### Results

The data analysis strategy was identical to that reported in Experiment 1 with one exception. The between-group factor contained three levels—Extended Training versus Limited Training versus No-Training. The mean rates of avoidance, approach, and competing behaviour in response to each GS during the generalisation tests are also illustrated in Supplementary Materials (Figures S3 to S5).

#### Manipulation checks

##### Category learning

MTS task training and testing trials were completed with a high level of accuracy (training accuracy >86%; testing accuracy >90%) (Table 2). This suggests that two artificial stimulus categories were established—CS1 was categorically related to GS1 and CS2 was categorically related to GS2. There was a non-significant group effect (Table 2).

**Table 2.** Response accuracy during category learning and competing behaviour learning stages in Experiment 2.

Accuracy	Total		Training group						Effect of group	
Category learning	*M*	*SD*	Extended		Limited		No training		*F*	*p*
			*M*	*SD*	*M*	*SD*	*M*	*SD*		
Training (%)	88.24	6.77	86.82	8.58	89.92	4.69	88.03	6.27	1.42	.25
Testing (%)	92.48	14.24	90.28	16.38	93.03	16.23	94.23	9.00	0.53	.59
Competing behaviour										
Training (%)	92.63	6.65	91.09	7.60	94.22	5.17	–	–	3.04	.09
Testing (%)	94.51	7.73	90.55	8.77	98.61	3.12	–	–	19.55	< .001

##### Avoidance learning

Avoidance was heightened for CS1 relative to CS2, *F*(1, 76) = 790.04, *p* < .001, $\eta_{p}^{2}=.91$ (Figure 4). There was a non-significant effect of group on avoidance, *F*(2, 76) = 2.92, *p* = .06, and a non-significant two-way interaction between group and stimulus, *F*(2, 76) = 1.62, *p* = .21. In addition, approach was heightened for CS2 relative to CS1, *F*(1, 76) = 989.87, *p* < .001, $\eta_{p}^{2}=.93$. There was a non-significant effect of group on approach, *F*(2, 72) = 2.48, *p* = .09, and a non-significant two-way interaction between stimulus and group, *F*(1, 76) = 1.16, *p* = .32 (Figure 4).

##### Differential reinforcement of competing behaviours

Competing behaviour training and testing trials were completed with a high level of accuracy (training accuracy >91%; testing accuracy >90%) (Table 2). This suggests that competing behaviours in response to CS1 and CS2 were learned. However, accuracy rates were higher in the Limited Training group (Table 2).

#### Outcome measures

All effects are described in Supplementary Materials (Tables S4 to S6).

##### Generalised avoidance

An RM-ANOVA examined the effect of stimulus, context, and group on avoidance. There was a significant three-way interaction between stimulus, context, and group, *F*(2, 73) = 6.04, *p* = .006, $\eta_{p}^{2}=.13$ (Table S4). The stimulus by context interaction was therefore examined separately across the groups. In the No-Training group, avoidance of GS1 relative to GS2 did not differ between the Novel and Acquisition contexts, *t*(25) = 1.44, *p* = .16 (Bonferroni-corrected α = .017) (Figure 5). This finding suggests that presenting the generalisation stimuli in the Novel context alone does not reduce generalised avoidance. Reductions in generalised avoidance were observed in the Extended and Limited Training groups. Avoidance of GS1 relative to GS2 was smaller in the Novel context than in the Acquisition context: *Limited Training, t*(26) = −3.16, *p* = .004; *Extended Training, t*(26) = −2.60, *p* = .017 (Figure 5). Thus, the differential reinforcement of competing behaviours led to a reduction in generalised avoidance in Novel contexts.

##### Competing behaviours

The No-Training group did not learn any competing behaviours, so were excluded from this analysis. An RM-ANOVA examined the effect of stimulus, context, and group on competing behaviours. There was a significant three-way interaction between stimulus, context, and group, *F*(1, 51) = 5.34, *p* = .025, $\eta_{p}^{2}=.01$ (Figure 5; Table S5). In the Extended Training group, a greater number of competing behaviours were observed in the Novel context relative to the Acquisition context, *F*(1, 26) = 14.23, *p* = .001, $\eta_{p}^{2}=.36$. However, in the Limited Training group, the number of competing behaviours did not differ between the Novel and Acquisition contexts, *F*(1, 25) = 1.63, *p* = .21. This could suggest that Extended Training increased the rate competing behaviours in Novel contexts.

##### Generalised approach

An RM-ANOVA examined the effect of stimulus, context, and group on approach. There was a significant interaction between stimulus and context, *F*(1, 76) = 4.65, *p* = .03, $\eta_{p}^{2}=.06$. Approach of GS2 relative to GS1 was lower in the Novel context than in the Acquisition context, *F*(1, 78) = 4.57, *p* = .04, $\eta_{p}^{2}=.06$. This was the case in all groups as indicated by the non-significant three-way interaction between stimulus, context, and group, *F*(2, 76) = 2.54 *p* = .09 (Figure 5). However, there was a significant interaction between stimulus and group, *F*(2, 76) = 6.28, *p* = .003, $\eta_{p}^{2}=.14$. Post hoc comparisons revealed that in the Novel context, the No-Training group approached GS2 more than the Extended Training group, *t*(51) = 5.19, *p* < .0001, and the Limited Training group, *t*(50) = 4.10, *p* < .0001 (Bonferroni-corrected α= .008). This implies that the differential reinforcement of competing behaviours led to a reduction in generalised approach in Novel contexts.

## General discussion

This study investigated an operant-based approach to reduce generalised avoidance. Avoidance in response to a threat-predictive stimulus was established in the Acquisition context. Some participants, but not others, then learned competing behaviours to the threat-predictive stimulus in different contexts. In a final test stage, generalised avoidance was lower in the groups that learned competing behaviours (Experiments 1 and 2). Specifically, stimuli that were similar to the threat-predictive stimulus elicited avoidance in the original threat Acquisition context and not in a Novel context. In the absence of competing behaviours, however, stimuli that were categorically related to a threat-predictive stimulus triggered avoidance in the Acquisition context and a Novel context (Experiment 2). These findings suggest that reinforcing new classes of competing behaviours may be an effective means to mitigate generalised avoidance in new contexts.

Generalised avoidance was lowered in Novel contexts even though the option to avoid was available. In contrast, experimental evidence suggests that standard fear extinction procedures have a limited impact on avoidance behaviour (Bravo-Rivera et al., 2014, 2015). These studies indicate, even after the extinction of Pavlovian fear responses, avoidance behaviour is common once the option to avoid is made available. For example, Vervliet and Indekeu (2015) delivered a brief electric shock to participants’ wrists after a threat-predictive stimulus (CS) appeared unless a specific key press was made. Avoidance was then blocked and the CS was presented in extinction. While fear responding was extinguished, avoidance returned when the key presses were re-presented. Although the option to avoid was available in the current experiments, there was no increase in avoidance in the Novel context. These findings suggest that reinforcing competing behaviours affords sustained reductions in avoidance, over-and-above what might be expected from fear extinction alone.

Previous research has focused on achieving global reductions in problematic avoidance with little consideration for the role of context (Bravo-Rivera et al., 2014, 2015; Claes et al., 2016; Gillan et al., 2014; Vervliet & Indekeu, 2015). The current studies highlight another option. Rather than erasing avoidance, it might be useful to encourage judicial avoidance strategies that are informed by situational details (Hofmann & Hay, 2018). Indeed, avoidance is an adaptive emotional regulation strategy depending on the context. Avoidance might be wise when a stranger begins to approach you on a dark street in an unsafe neighbourhood, but it is less adaptive when a stranger approaches you at a work event. By reinforcing competing behaviours in new contexts, we observed an emergent pattern of generalised avoidance that was sensitive to contextual details. This was characterised by an increase in avoidance in original threat-relevant context but not in new contexts that were never previously associated with threat.

The specific role of competing behaviours was investigated using a between-groups design. An Extended Training group learned two classes of competing behaviours in two different contexts, whereas a Limited Training group learned only one additional class of competing behaviours. It was expected that the former training condition would result in great reductions in generalised avoidance. But this was not the case. Generalised avoidance was reduced in new contexts, and this did not differ between the two training groups. It could be suggested that simply presenting the Novel context leads to reductions in generalised avoidance. However, there were no reductions in generalised avoidance observed for a group that learned no competing behaviours. One possibility is that training of even more competing behaviours would result in greater reductions in generalised avoidance. Future research could therefore include an additional group who learn a greater number of competing behaviours across a greater number of contexts.

These studies focused on the generalisation of avoidance within artificially created categories. This literature almost exclusively focused on the perceptual generalisation of avoidance between stimuli that are perceptually similar (Lissek, 2012; Lissek et al., 2008; Lommen et al., 2010). Yet in the real world, cases of perceptually generalised avoidance are not always evident. In anxiety disorders, for example, ever-growing networks of physically dissimilar stimuli trigger avoidance because of their category membership (i.e., “things that are unsafe”). This suggests that category-level information can be recruited during avoidance learning such that an entire category is associated with threat (Bennett, Vervoort, et al., 2015; Dunsmoor & Murphy, 2015; Dymond et al., 2015; Meulders & Bennett, 2017). This form of generalisation is highly problematic because category-based relations have substantial scope; they are abstract and not restricted by physical form. Therefore, the category-based generalisation of avoidance is worthy of further investigation.

Some procedural limitations should be mentioned. First, this study did not employ any physiological or self-report measurements of fear. These measures are common in conditioning research and confer information about the subjective emotional experience (Boddez et al., 2013; Lonsdorf et al., 2017). However, our focus was on avoidance which is not always concordant with these measures (Rachman, 1990; Rescorla & Lolordo, 1965). Second, the avoidance behaviour in this study was low-cost and did not precipitate negative consequences. Real-world avoidance tends to be costly as it interferes with valued routines. An important next step will be to extend our findings to high-cost avoidance behaviours. However, our study is still clinically relevant. Patients with anxiety disorder often rely on subtle and low-cost safety behaviours (e.g., tapping the fuselage of a plane for good luck or keeping prescription pills nearby; Meulders et al., 2016; Vervliet & Indekeu, 2015). Finally, the avoidance and competing behaviours in this study were well matched in terms of the effort they require. However, in clinical settings, it is normally easier for individuals to engage in their long-standing avoidance tendencies than it is to develop newer and more appropriate behaviours. In this way, the relative effort that competing behaviours require is likely to be an important determinant of therapeutic change. This study can provide an experimental framework for future studies to explore the role of competing behaviour accessibility and effort.

Interestingly, the prototype intervention examined here parallels a therapeutic strategy known as cognitive defusion, as described by Acceptance and Commitment Therapy (ACT; Hayes et al., 2006, 2012). Cognitive fusion is described as a trans-diagnostic process in which there is inefficient contextual control over the response functions of key threat stimuli. Cognitive defusion exercises aim to disrupt category-based generalisation of fear/avoidance by re-establishing contextual control (Assaz et al., 2018). In what is referred to as the “milk, milk, milk exercise,” for example, clients repeat a target symbolic stimulus aloud (e.g., saying the word “panic” over-and-over; see Masuda & Hayes, 2004). Across repetitions, response functions such as auditory features of the stimulus, sensory–motor facets of pronunciation, and an increasing variety of emotional responses change in relative salience such that a formerly dominant problematic response function (e.g., avoidance) changes in probability. These experiential exercises evidently reduce problematic emotional responses to target words (Masuda et al., 2004, 2009; Tyndall et al., 2017). However, there is little experimental evidence to suggest that the therapeutic change is driven by disruptions in category-based generalisation, as claimed by ACT (Assaz et al., 2018). Indeed, there is a paucity of experimental research examining if, and how, disruptions in category-based generalisation can even be achieved (Vlaeyen, 2014). This study, however, demonstrates that the category-based generalisation of avoidance is disrupted by reinforcing competing behaviours to threat-predictive CSs. This finding provides some insight into a potential mechanism through which cognitive defusion could operate.

## Conclusion

Strategies to reduce generalised avoidance are under-investigated. Even less studied are ways to reduce the category-based generalisation of avoidance. We examined the role of an operant-based approach which represents a novel way of mitigating generalised avoidance. This study is among the first to successfully control the generalisation of category-based avoidance under laboratory conditions and, as far as we are aware, it is the first to demonstrate a method that may be adapted for use in clinical contexts to reduce the category-based generalisation of avoidance.

## Supplemental Material

supplementary_materials – Supplemental material for Transitions from avoidance: Reinforcing competing behaviours reduces generalised avoidance in new contexts Click here for additional data file. Supplemental material, supplementary_materials for Transitions from avoidance: Reinforcing competing behaviours reduces generalised avoidance in new contexts by Marc P Bennett, Bryan Roche, Simon Dymond, Frank Baeyens, Bram Vervliet and Dirk Hermans in Quarterly Journal of Experimental Psychology
