# Supplementary material for: Transitions from avoidance: Reinforcing competing behaviours reduces generalised avoidance in new contexts
Source: Q J Exp Psychol (Hove). 2020 Aug 21;73(12):2119–31. doi: 10.1177/1747021820943148 (PMC7672781; doi:10.1177/1747021820943148)
Supplement: supplementary_materials – Supplemental material for Transitions from avoidance: Reinforcing competing behaviours reduces generalised avoidance in new contexts [file supplementary_materials.docx]

**Transitions from avoidance: Reinforcing competing behaviors reduces generalized avoidance in new contexts**

**Supplementary Materials**

Marc P. Bennett, Bryan Roche, Simon Dymond, Frank Baeyens, Bram Vervliet & Dirk Hermans

Correspondence for this article should be addressed to Dr. Marc Bennett, Medical Research Council- Cognition and Brain Sciences Unit, 15 Chaucer Road, Cambridge, CB2 7EF

E-mail: marc.bennett@mrc-cbu.cam.ac.uk

**Task instructions**

Written instructions appeared on the computer screen at the different experimental stages. Instructions were presented in Dutch but an English translation is as follows:

**MTS training and testing.** *In this task you will see a set of 4 symbols appear on the screen: one at the top and three at the bottom (one on the left, one in the middle and one on the right). Your task is to select the correct symbol at the bottom of the screen by pressing the numeric buttons 1, 2 and 3. During the phase you will receive feedback with each choice. Later you will not receive feedback every time. There is always a correct answer. You should give as many correct answers as possible. During the first part, you might find the task easy and it will be tempting to stop paying attention. However, the difficulty could increase and choosing the correct symbols in the later part of this phase will depend on the knowledge you collect during the first parts. Things you learn in this part of the experiment may be important later.*

**Pavlovian conditioning.** *In this part of the experiment, you will learn different responses to different events and these can have different consequences. Please note that the color of the screen may change and that this may contain relevant information for carrying out your task. In this next step, some items will be followed by unpleasant photos and sounds. You will be awarded points for other items. Your job is to be attentive and learn which items bring which consequences.*

**Avoidance and approach.** *Now, you can win points by pressing <enter> key at the right time. In addition, you can also avoid unpleasant photos and sounds by pressing <space> at the right time. Your task is to get as many points as possible and avoid as many unpleasant stimuli as possible.*

**Competing behaviors.** *[Opening] During this next stage, your task is to learn new responses. [Context-1] You now have to learn when to press 'T' and when to press 'P'. [Context-2] You now have to learn when to press 'W' and when to press 'X'. [Accuracy check: Extended training group] Your job now is to put together everything you have learned. Sometimes you have to press T or P, sometimes you have to press W or X, and sometimes you have to press enter or space. [Accuracy check: Limited training group] Your job now is to put together everything you have learned. Sometimes you have to press T or P, and sometimes you have to press enter or space.*

**Generalization test.** *Your job now is to make the most appropriate responses: that is, the responses you think are best.*

**IAP identifiers**

Twelve images of body mutilations were taken from the International Affective Picture System (IAPS) and shown for 3 s (1024x768 pixels) in combination with aversive sound. These images included IAP #3000, #3010, #3030, #3051, #3062, #3063, #3064, #3080, #3100, #3102, #3130, #3150.

**Generalized Avoidance, Approach and Competing Behavior**


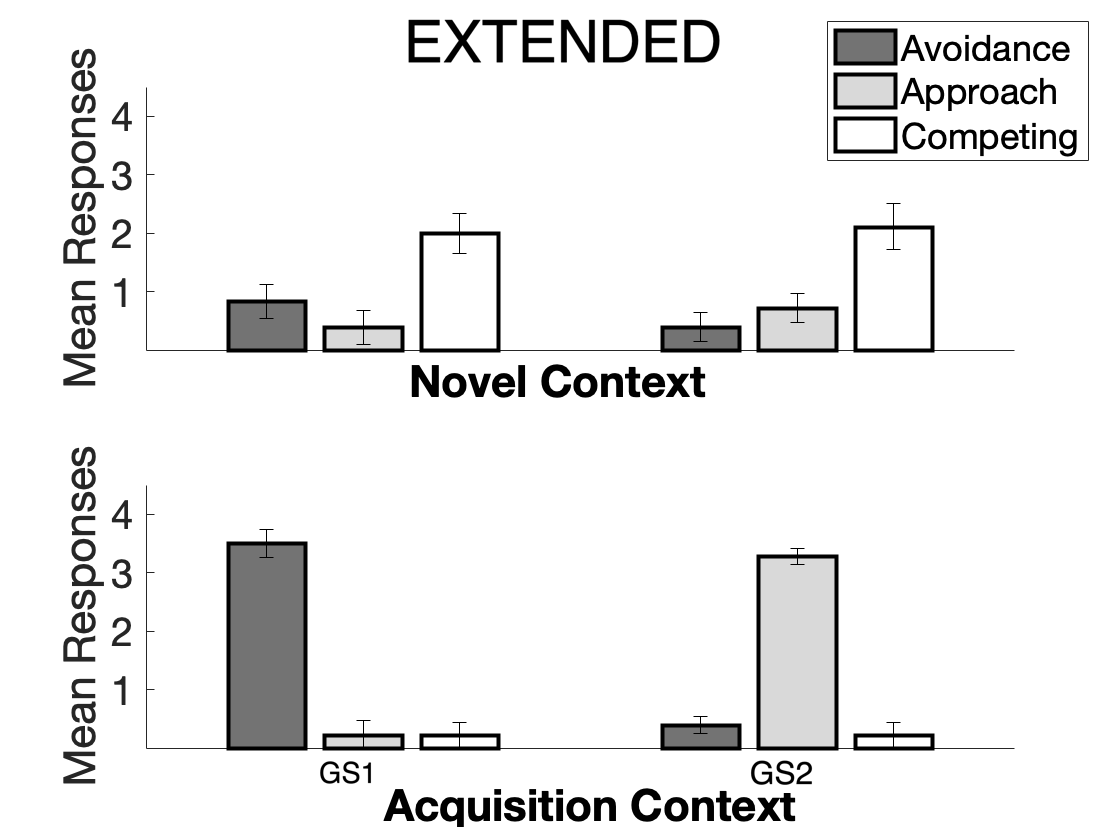


Figure S1. Mean avoidance, approach and competing behaviours in response to GS1 and GS2. This figure illustrates mean responses for the Extended Training group in both contexts during Experiment 1. Error bars indicated standard error.

**
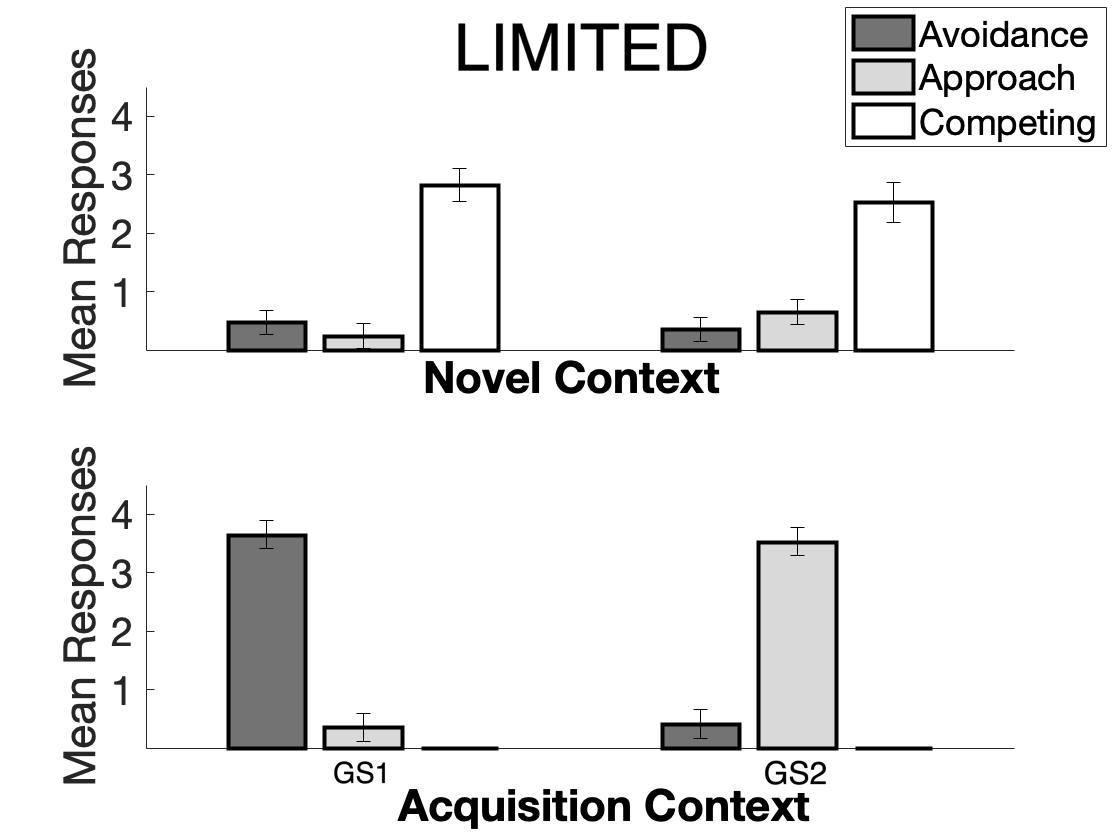
**

Figure S2. Mean avoidance, approach and competing behaviours in response to GS1 and GS2. This figure illustrates mean responses for the Limited Training group in both contexts during Experiment 1. Error bars indicated standard error.

**
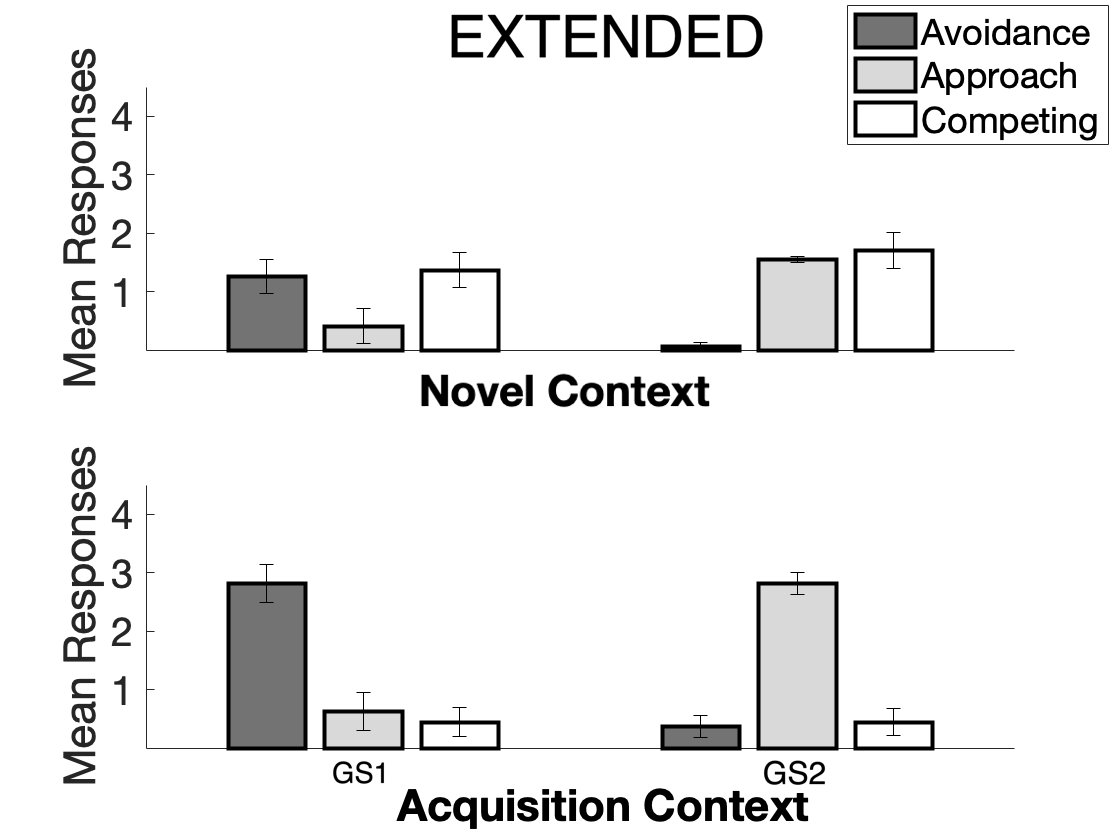
**

Figure S3. Mean avoidance, approach and competing behaviours in response to GS1 and GS2. This figure illustrates mean responses for the Extended Training group in both contexts during Experiment 2. Error bars indicated standard error.

**
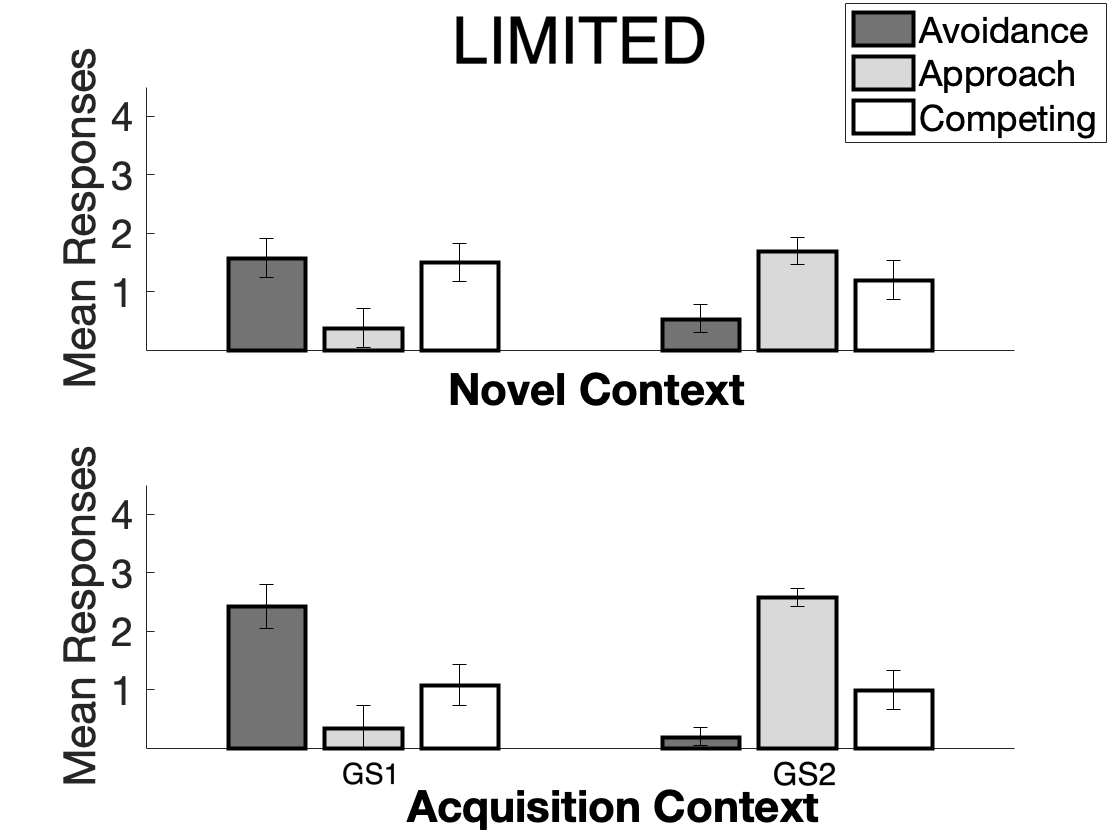
**

Figure S4. Mean avoidance, approach and competing behaviours in response to GS1 and GS2. This figure illustrates mean responses for the Limited Training group in both contexts during Experiment 2. Error bars indicated standard error.

**
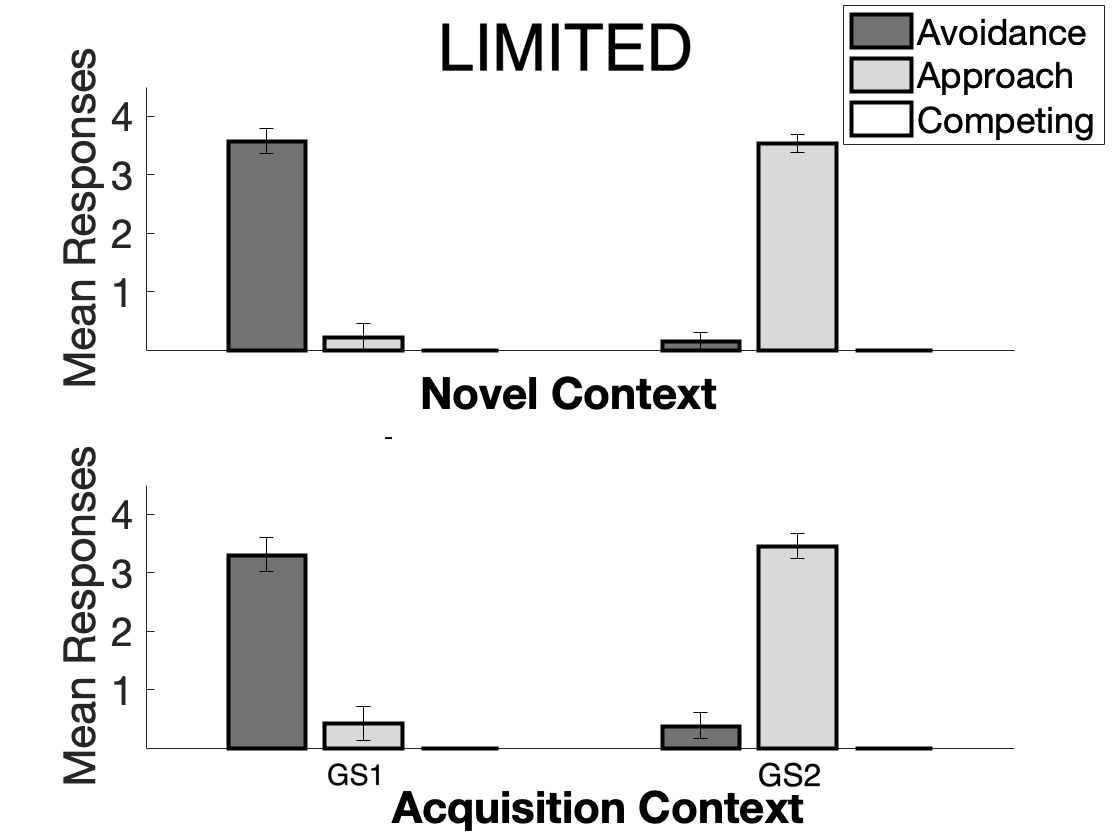
**

Figure S5. Mean avoidance, approach and competing behaviours in response to GS1 and GS2. This figure illustrates mean responses for the Control group in both contexts during Experiment 2. Error bars indicated standard error.

**Main effects of the RM-ANOVA**

A series of repeated measures (RM)-ANOVA were calculated in Experiment 1 and Experiment 2. The aim was to examine the effect of stimulus (GS1 vs. GS2), context (Acquisition-context vs. Novel-context) and group (Extended vs. Limited [vs. No] Training) on generalized avoidance. Similar models were also calculated with approach and competing behaviors included as the dependent variables. Below, the main effects of each RM-ANOVA are described.

**Experiment 1**

| Table S1. Main effects from a RM-ANOVA investigating the effect of stimulus, context and group on generalized avoidance | | | | | | |
| --- | --- | --- | --- | --- | --- | --- |
|  |  |  |  |  |  |  |
| *Effects* |  |  |  |  |  |  |
|  |  | **F** | **DF** | | **p** | **ηp2** |
| stimulus |  | 58,76 | 1, 33 | | <.001 | 0,64 |
| group |  | 0,21 | 1, 33 | | .65 | 0,006 |
| stimulus * group |  | 0,05 | 1, 33 | | .824 | 0,002 |
| context |  | 101,45 | 1, 33 | | <.001 | 0,755 |
| context * group |  | 0,94 | 1, 33 | | .339 | 0,028 |
| stimulus * context |  | 100.80 | 1, 33 | | <.001 | 0,753 |
| stimulus * context * group |  | 0,61 | 1, 33 | | .439 | 0,018 |

| Table S2. Main effects from a RM-ANOVA investigating the effect of stimulus, context and group on competing behaviors | | | | | | |
| --- | --- | --- | --- | --- | --- | --- |
|  |  |  |  |  |  |  |
| *Effects* |  |  |  |  |  |  |
|  |  | **F** | **DF** | | **p** | **ηp2** |
| stimulus |  | 0,2 | 1, 33 | | .66 | 0,01 |
| group |  | 0,77 | 1, 33 | | 0,39 | 0,02 |
| stimulus * group |  | 0.10 | 1, 33 | | .33 | 0,03 |
| context |  | 69,47 | 1, 33 | | <.001 | 0,68 |
| context * group |  | 2,43 | 1, 33 | | .13 | 0,07 |
| stimulus * context |  | 0.20 | 1, 33 | | .66 | 0,01 |
| stimulus * context * group |  | 0,96 | 1, 33 | | .33 | 0,03 |

| Table S3. Main effects from a RM-ANOVA investigating the effect of stimulus, context and group on generalized approach | | | | | | |
| --- | --- | --- | --- | --- | --- | --- |
|  |  |  |  |  |  |  |
| *Effects* |  |  |  |  |  |  |
|  |  | **F** | **DF** | | **p** | **ηp2** |
| stimulus |  | 80,53 | 1, 33 | | <.001 | 0,709 |
| group |  | 0.10 | 1, 33 | | .759 | 0,003 |
| stimulus * group |  | 0,07 | 1, 33 | | .799 | 0,002 |
| context |  | 69,66 | 1, 33 | | <.001 | 0,679 |
| context * group |  | 0.90 | 1, 33 | | .351 | 0,026 |
| stimulus * context |  | 56.90 | 1, 33 | | <.001 | 0,633 |
| stimulus * context * group |  | 0,003 | 1, 33 | | .954 | 0 |

**Experiment 2**

| Table S4. Main effects from a RM-ANOVA investigating the effect of stimulus, context and group on generalized avoidance | | | | | | |
| --- | --- | --- | --- | --- | --- | --- |
|  |  |  |  |  |  |  |
| *Effects* |  |  |  |  |  |  |
|  |  | **F** | **DF** | | **p** | **ηp2** |
| stimulus |  | 121,96 | 1, 76 | | <.001 | 0,62 |
| group |  | 11.30 | 2,76 | | <.001 | 0,23 |
| stimulus * group |  | 5,85 | 2,76 | | .004 | 0,13 |
| context |  | 13,08 | 1,76 | | .001 | 0,15 |
| context * group |  | 7,03 | 2,76 | | .002 | 0,16 |
| stimulus * context |  | 7.70 | 1,76 | | .007 | 0,09 |
| stimulus * context * group |  | 6.00 | 2,76 | | .004 | 0,14 |

| Table S5. Main effects from a RM-ANOVA investigating the effect of stimulus, context and group on competing behaviors | | | | | | |
| --- | --- | --- | --- | --- | --- | --- |
|  |  |  |  |  |  |  |
| *Effects* |  |  |  |  |  |  |
|  |  | **F** | **DF** | | **p** | **ηp2** |
| stimulus |  | 0,02 | 1, 51 | | .96 | <0.001 |
| group |  | 0,41 | 1, 51 | | .53 | 0,01 |
| stimulus * group |  | 2.80 | 1, 51 | | .10 | 0,05 |
| context |  | 6,02 | 1,51 | | .02 | 0,12 |
| context * group |  | 2,01 | 1, 51 | | .16 | 0,04 |
| stimulus * context |  | 0,33 | 1, 51 | | .57 | 0,01 |
| stimulus * context * group |  | 5,34 | 1, 51 | | .03 | 0.10 |

| Table S5. Main effects from a RM-ANOVA investigating the effect of stimulus, context and group on competing behaviors | | | | | | |
| --- | --- | --- | --- | --- | --- | --- |
|  |  |  |  |  |  |  |
| *Effects* |  |  |  |  |  |  |
|  |  | **F** | **DF** | | **p** | **ηp2** |
| stimulus |  | 0,02 | 1, 51 | | .96 | <0.001 |
| group |  | 0,41 | 1, 51 | | .53 | 0,01 |
| stimulus * group |  | 2.80 | 1, 51 | | .10 | 0,05 |
| context |  | 6,02 | 1,51 | | .02 | 0,12 |
| context * group |  | 2,01 | 1, 51 | | .16 | 0,04 |
| stimulus * context |  | 0,33 | 1, 51 | | .57 | 0,01 |
| stimulus * context * group |  | 5,34 | 1, 51 | | .03 | 0.10 |
